# An in-depth analysis of UAV path planning, including procedures, algorithms, optimization models, and emerging challenges

**DOI:** 10.1016/j.mex.2026.103826

**Published:** 2026-02-17

**Authors:** Muhammad Nafees, Tamkeen Syeda, Amjad Ali, Hira Farman, Muhammad Tayyab

**Affiliations:** a Computer Science & IT, Karachi Institute of Economics and Technology, Karachi Sindh, Pakistan; b Computer Science & IT, Iqra University, Karachi Sindh, Pakistan

**Keywords:** Multi-UAV coordination, Optimization algorithms, UAV path planning, Problem models, Unmanned aerial vehicles (UAVs)

## Abstract

Unmanned aerial vehicles (UAVs) have emerged as valuable assets in modern surveillance, environmental monitoring, disaster response, and delivery systems. Their autonomy is built around effective path planning, which provides safe, energy-efficient, and goal-oriented navigation in complex, dynamic environments. This paper provides a comprehensive overview of UAV path planning research, encompassing classification techniques, heuristic and metaheuristic algorithms, and underlying problem models. It emphasizes significant contributions from traditional, hybrid, and AI-driven techniques while carefully highlighting upcoming issues, such as real-time processing, multi-UAV coordination, energy limitations, and environmental concerns. The study provides a detailed assessment of the strengths and weaknesses of significant algorithms, allowing for the identification of research gaps and future initiatives. This paper is an invaluable resource for researchers and practitioners seeking to create robust, scalable, and adaptive UAV route planning systems appropriate for a wide range of mission-specific and real-world applications.

## Review Highlights

• **Comprehensive Overview** – The paper surveys UAV path planning research, covering classification techniques, traditional, heuristic / metaheuristic, hybrid, and AI-driven algorithms, along with various problem models.
• **Key Insights** – It evaluates the strengths and weaknesses of existing methods, highlighting their effectiveness in ensuring safe, energy-efficient, and adaptive UAV navigation in complex environments.
• **Future Directions** – The study identifies research gaps and emphasizes challenges such as real-time processing, multi-UAV coordination, energy efficiency, and environmental concerns to guide future innovations.

## Graphical abstract

**Figure:**
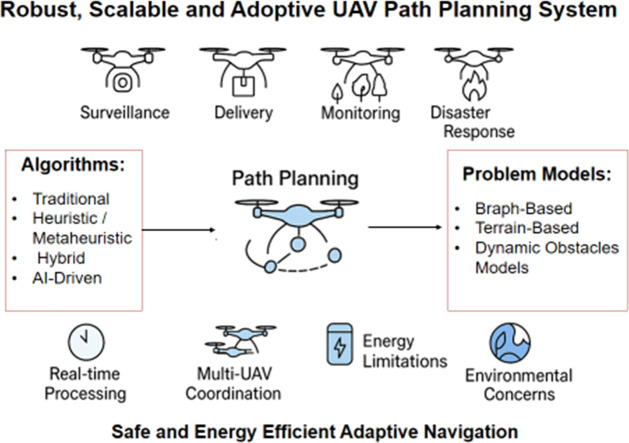

## Specifications table

**Table:** 

**Subject area**	Computer Science
**More specific subject area**	UAV Path Planning
**Name of the reviewed methodology**	Research Questions and Survey Research
**Keywords**	Multi-UAV Coordination, Optimization Algorithms, UAV Path Planning, Problem Models, Unmanned Aerial Vehicles (UAVs)
**Resource availability**	Not applicable
**Review question**	RQ-1: Which techniques have been used so far for UAV path planning? RQ-2: Which algorithms have been applied in UAV path planning? RQ-3: What types of problem models have been addressed in UAV path planning, such as obstacle avoidance, energy efficiency, and multi-objective planning? RQ-4: What emerging challenges are being faced in UAV path planning? RQ-5: How UAV path planning algorithms and problem models can effectively handle operational challenges and adapt to mission-specific environments?

## Background

Drones, also known as Unmanned Aerial Vehicles (UAVs), have become indispensable tools in a multiplicity of applications. Planning effective and collision-free routes in a variety of sometimes unexpected situations is essential to UAV operational success. Calculating an ideal or nearly ideal route from a starting point to a destination while taking mission-specific goals, environmental restrictions, and UAV dynamics into account is known as path planning. The complexity of UAV missions has grown as their deployment expands across domains, necessitating the development of increasingly sophisticated, flexible, and scalable route planning algorithms. To address these issues, researchers have proposed a range of methods, including sophisticated metaheuristics, machine learning-based models, and traditional optimization approaches.

Robotic applications, or UAVs, have been widely applied in various fields, including industrial [1], agricultural [2], surveillance [3], search and rescue [4], environmental monitoring [5], and target tracking [6]. Lightweight, small-sized UAVs are being rapidly used for recreational, hobby, and industrial purposes [7,8]. UAVs and the sensors they can carry have advanced significantly in recent years due to rapid technical development, enabling them to cover a wide range of applications [9]. UAVs are a valuable and effective tool for enhancing disaster situational awareness among responders [10].

It is the most crucial aspect of vehicle navigation, with the goal of finding the quickest and most collision-free path from the present robot's location to a specific objective. In many real-world navigation missions, a UAV's flight environment may be dynamic, with obstacles and hazards changing over time. In these harsh conditions, the collision-free path planning task becomes increasingly difficult and expensive, posing a significant challenge to the UAV's control and autonomous navigation system. In dynamic situations, a UAV drone responds to the position and speed of shifting obstacles.

The drone must adhere to these dynamic limitations and respond quickly to such changes. As a result, planning algorithms utilized in this formalism must demonstrate outstanding performance, particularly in terms of execution time and processing speed [11].. Multiple UAVs must work together continuously to plan efficient paths. The process of planning a course to navigate is considered a fundamental duty for drones of various varieties. The task assignment and coordination procedure is streamlined with the use of a base station or Ground Control Station (GCS) for managing Unmanned Aerial Vehicles (UAVs). UAV path planning can be performed both online and offline. Path planning in UAVs can be accomplished using meta-heuristic optimum path planning algorithms, which give an ideal path for navigation during a surveillance mission [12].

## Method details

Domestic and international scholars have proposed many techniques for path planning of UAVs or robots [13], such as Dijkstra Algorithm [42], A* Algorithm [16], Ant Colony Optimization (ACO) [26], Artificial Bee Colony (ABC) [58], Genetic Algorithm (GA) [54], Bat Algorithm (BA) [33], Cuckoo Search (CS) [17], Particle Swarm Optimization (PSO) [32], Grey Wolf Optimizer (GWO) [44], Differential Evolution (DE) [47], etc. However, when the target and the obstacle are close together, the algorithm may fall into a local optimum, and the optimal flyable path is not guaranteed. In recent years, the population-based evolutionary algorithm has made significant development.

The primary goal of path search, a crucial component of path planning, is to develop a planning algorithm that effectively navigates the path. Reliable and fair path planning helps assure UAV safety as well as mission success in a complex environment. However, there are numerous limitations to UAV path planning, including computational complexity, wireless interference, battery constraints, and scalability in complex environments, as well as obstacle avoidance, communication constraints, and swarm coordination [51].

In order to systematically investigate the current state-of-the-art and identify potential research directions, this study is structured around the following research questions:

**RQ-1: Which techniques have been used so far for UAV path planning?**
**RQ-2: Which algorithms have been applied in UAV path planning?**
**RQ-3: What types of problem models have been addressed in UAV path planning, such as obstacle avoidance, energy efficiency, and multi-objective planning?**
**RQ-4: What emerging challenges are being faced in UAV path planning?**
**RQ-5: How UAV path planning algorithms and problem models can effectively handle operational challenges and adapt to mission-specific environments?**

This paper provides a comprehensive analysis of UAV path planning strategies. It begins with an introduction that explains the significance and scope of UAV path planning. The study's significant contributions are then highlighted to underline the work's uniqueness and impact. A thorough Literature Review follows, summarizing recent advances and research gaps. The Classification of UAV Path Planning Techniques section categorizes existing methodologies. The UAV Path Planning Algorithms section then looks into notable algorithms before moving on to an investigation of various Problem Models used in the domain. The Emerging Challenges section addresses outstanding challenges and technical barriers. Finally, the Discussion interprets significant findings in light of previous research, while the Conclusion and Future Directions provide a summary and identify intriguing areas for future investigation.

## Major contributions of the study

This survey paper presents a comprehensive and structured review of existing literature on UAV route and path planning, focusing on both algorithmic techniques and problem formulations. The significant contributions of this study are as follows:

### Taxonomy of path planning procedures

The paper classifies a wide range of path planning techniques used in UAV research, including classical graph-based methods (e.g., A*, Dijkstra), sampling-based methods (e.g., RRT, PRM), and advanced heuristic/metaheuristic techniques such as Tabu Search, Genetic Algorithms, Particle Swarm Optimization, Ant Colony Optimization, and Artificial Bee Colony algorithms, among others.

### Review of applied algorithms and their capabilities

A comprehensive comparative examination of how several algorithms have been applied to UAV path planning problems is presented, with an emphasis on adaptability, computational efficiency, convergence behavior, and applicability in various environmental contexts.

### Classification of problem models

The survey investigates the many issue models used in UAV path planning, such as obstacle avoidance, energy-efficient routing, terrain-aware navigation, real-time re-planning, and multi-objective optimization. It demonstrates how several algorithms are designed to address these distinct difficulties.

### Mapping applications across domains

The study examines the application of UAV path-planning approaches in various fields, including industry [1], agriculture [2], surveillance [3], search and rescue [4], environmental monitoring [5], and target touring [6], among others. It investigates the individual path planning objectives for each application, such as coverage maximization, time efficiency, and target tracking.

### Identification of emerging challenges and future directions

The paper identifies ongoing challenges in the field, such as handling dynamic obstacles, integrating machine learning for adaptive planning, designing standardized benchmarking frameworks, and ensuring real-time performance in multi-UAV systems. These insights offer a foundation for guiding future research efforts.

## Literature review

Several strategies have been developed to create an optimal UAV path for various missions [2]. Several approaches have been devised and deployed to address the general challenges associated with path planning [3. This section summarizes existing work on the motion framework [4]. It is not easy to control UAVs to autonomously follow moving targets while meeting mission requirements, such as maintaining continuous coverage or keeping a non-deviant camera aim point in the face of wind [7].

To ensure a seamless mission, it is critical to prioritize safety and reliability. That is, it is required to find the quickest way to the objective while avoiding impediments [8].

Over the past two decades, UAV path planning has been a topic of significant research driven by the growing demand for autonomous aerial navigation in both military and civilian applications. Numerous algorithms and optimization models have been proposed to enhance path efficiency, reduce energy consumption, facilitate obstacle avoidance, and facilitate real-time decision-making.

Numerous previous studies have employed solo algorithms, which are individual optimization or search strategies, to address UAV path planning challenges such as obstacle avoidance, optimal routing, energy conservation, and mission completion, among others. These algorithms encompass both classical methods and nature-inspired approaches. Each of these algorithms was tested individually in unique circumstances to determine their effectiveness under different constraints. In this survey, we summarize the utilization of solo algorithms as described in previous studies. A complete summary is presented in the table below, highlighting their uses, benefits, and limits in various UAV planning scenarios.

Table 1 shows that due to their adaptability in managing intricate and high-dimensional search spaces, metaheuristic and swarm intelligence-based algorithms have dominated contemporary UAV path planning research. Additionally, it is clear that the majority of studies have distance and obstacle avoidance as their main goals, but energy-aware and communication-constrained planning receive relatively less attention, pointing to possible research needs.

**Table 1:** Summary of existing solo UAV path planning Algorithms.

References	Existing Algorithms
[1,4,42,43,61]	Dijkstra Algorithm
[4,16,40,[42], [43], [44],^47^,^59^]	A* Algorithm
[3,12,26,30,[45], [46], [47], [48], [49],^51^,^65^]	Ant Colony Optimization (ACO)
[2,3,12,30,45,46,49,58]	Artificial Bee Colony (ABC)
[1,4,19,21,30,[45], [46], [47], [48], [49],^54^]	Genetic Algorithm (GA)
[3,12,30,33,45,49]	Bat Algorithm (BA)
[30,45]	Cuckoo Search (CS)
[1,3,4,15,30,[45], [46], [47],^49^,^63^]	Particle Swarm Optimization (PSO)
[12,30,[44], [45], [46],^49^]	Grey Wolf Optimizer (GWO)
[[45], [46], [47],^49^,^64^]	Differential Evolution (DE)
[3,30,45,46]	Firefly Algorithm (FA)
[45,46]	Simulated Annealing (SA)
[30,49]	Sine Cosine Algorithm (SCA)
[2,58]	Hill Climbing
[8,42]	RRT

Table 2 shows that the research currently in publication places a great deal of attention on static and known contexts, with very few studies addressing highly dynamic or uncertain circumstances. Furthermore, hybrid techniques are being used more frequently to strike a compromise between computing efficiency and flexibility, especially in contexts with plenty of obstacles.

**Table 2:** Comprehensive study On UAV Path Planning Algorithms.

Ref	A*	ABC	ACO	Dijkstra	BA	CS	GA	FA	DE	GWO	PSO	CPSO	HC	SCA	DQL	DTO	BAS	SA	DNN	RRT
[1]				✓			✓				✓				✓					
[2]		✓											✓							
[3]		✓	✓		✓			✓			✓									
[4]	✓			✓			✓				✓									
[5]																				
[6]																				
[7]																				
[8]																				✓
[9]																				
[10]																				
[11]																				
[12]		✓	✓		✓					✓										
[13]																				
[14]																				
[15]											✓									
[16]	✓																			
[17]																				
[18]																				
[19]							✓													
[20]																				
[21]							✓													
[22]																				
[23]																				
[24]												✓								
[25]												✓								
[26]			✓																	
[27]																				
[28]																				
[29]																				
[30]		✓	✓		✓	✓	✓	✓		✓	✓			✓		✓				
[31]																	✓			
[32]												✓								
[33]					✓															
[34]																				
[35]																				
[36]																				
[37]																			✓	
[38]																				
[39]																				
[40]	✓																			
[41]																				
[42]	✓			✓																✓
[43]	✓			✓																
[44]	✓									✓										
[45]		✓	✓		✓	✓	✓	✓	✓	✓	✓							✓		
[46]		✓	✓				✓	✓	✓	✓	✓							✓		
[47]	✓		✓				✓		✓		✓									
[48]			✓				✓													
[49]		✓	✓		✓	✓	✓		✓	✓	✓			✓				✓		
[50]																				
[51]																				
[52]																				
[53]																				
[54]							✓													
[55]																				
[56]																				
[57]																				
[58]													✓							
[59]	✓																			
[60]																				
[61]				✓																
[62]	✓																			
[63]											✓									
[64]									✓											
[65]			✓																	
[66]	✓		✓								✓									✓
**Our Study**	**✓**	**✓**	**✓**	**✓**	**✓**	**✓**	**✓**	**✓**	**✓**	**✓**	**✓**	**✓**	**✓**	**✓**	**✓**	**✓**	**✓**	**✓**	**✓**	**✓**

A diverse set of standalone algorithms has been widely used in UAV route planning to address issues such as obstacle avoidance, trajectory optimization, coverage maximization, and energy efficiency. Dijkstra and A* are two of the most widely used classical algorithms, notable for their deterministic shortest-path search capabilities in grid- and graph-based contexts. These algorithms have been utilized in various research studies [1] for Dijkstra and [4] for A* due to their simplicity and optimality in structured contexts.

One of the most popular metaheuristic and swarm intelligence techniques is the Ant Colony Optimization (ACO) algorithm [12]. ACO's distributed and adaptive properties, inspired by ant foraging behavior, have resulted in effective performance in dynamic and complicated terrain. Similarly, the Artificial Bee Colony (ABC) method [12] and the Genetic method (GA) [21] have been intensively researched due to their high global search capability and flexibility in multi-objective optimization.

Other solo algorithms with notable applications include Particle Swarm Optimization (PSO) [32], known for its balance of exploration and exploitation, and the Bat Algorithm (BA) [33], which utilizes echolocation principles to facilitate path finding. Techniques such as Cuckoo Search (CS) [45] and Grey Wolf Optimizer (GWO) [49] have been employed in high-dimensional spaces due to their simplicity and convergence efficiency.

Differential Evolution (DE), Firefly Algorithm (FA), Simulated Annealing (SA), and Sine Cosine Algorithm (SCA) are four less popular yet successful methods that have been investigated in recent UAV-related studies for their ability to escape local optima in nonlinear search spaces. In addition, bio-inspired models such as the Whale Optimization Algorithm (WOA) [12,49] and classical techniques such as Hill Climbing (HC) [2,58] and Rapidly Exploring Random Trees (RRT) [8,42] have been used in niche scenarios requiring fast reactivity and exploration in unknown environments.

Overall, the studied literature indicates that while solo algorithms offer potential solutions, their performance is heavily influenced by the individual problem model, terrain conditions, and mission constraints. The preceding table contains specific references that show how each algorithm has been separately implemented in UAV route and path planning.

In addition to solo algorithmic approaches, numerous recent studies have investigated the use of hybrid algorithms to overcome the limits of single methodologies in UAV path planning. Hybrid approaches combine the strengths of two or more algorithms, such as merging a metaheuristic optimizer with a predictive control model or combining nature-inspired algorithms and fuzzy logic, to increase adaptability, solution accuracy, and computational efficiency. These techniques are instrumental in dynamic, unpredictable, or multi-objective settings. In this review, we present a summary of the primary hybrid strategies used in past research, including their components, application fields, and performance benefits. A detailed overview of hybrid algorithms used in UAV path planning is provided in the table below.

Table 3 shows that hybrid combinations of classical planners with metaheuristic or learning-based approaches are preferred in recent research trends. These combinations are mostly used to get over drawbacks like the trapping of local optima and the limited flexibility of standalone techniques. However, because of training complexity and computing limitations, completely autonomous learning-based frameworks are still largely unexplored.

**Table 3:** Summary of existing hybrid UAV path planning Algorithms.

References	Hybrid UAV Path Planning Algorithms
[2,55,58]	Hill Climbing + Artificial Bee Colony (HABC)
[13]	VAINDIWPSO and IC-VAINDIWPSO
[17]	DFSCSO (Dispersal Foraging Strategy + Cuckoo Search)
[19]	GA-QL (Genetic Algorithm + Q-Learning)
[21]	CPSO
[24,25]	Improved SABAS (Self-Adaptive Beetle Antennae Search)
[31]	Bat Algorithm + Asymmetrical Weighted Variational Method
[33]	GMOPSO-QL (Gaussian MOPSO + Q-Learning)
[34]	NN + TLBO (Neural Network + Teaching-Learning-Based Optimization)
[37]	GWO + A*
[44]	A* + DWA (Dynamic Window Approach)
[47]	AD3QN (Advanced Deep Double Dueling Q-Network)
[50]	ACO + PSO, GA + ACO
[51]	HT-PSO (Hyperbolic Tangent PSO)
[52]	LODBO (Landmark-guided Optimized Dung Beetle Optimization)
[53]	GA + Blockchain + Trust Model
[54]	Hybrid K-means + Local & Global TSP

Hybrid algorithms have emerged as an effective way to enhance the performance and reliability of UAV path planning by integrating the complementary qualities of different techniques. Several research have proposed several hybrid models to address the constraints of single algorithms in dynamic, unpredictable, and multi-objective settings.

For example, HABC [2,55], and [58] improve global search with local fine-tuning capabilities. Similarly, VAINDIWPSO and its improved chaotic counterpart, IC-VAINDIWPSO [13], were developed to balance exploration and exploitation dynamically. DFSCSO [17] is another bio-inspired hybrid that aims to increase convergence speed and solution quality.

Learning-based hybrids have also gained popularity, such as GA-QL [19,21], and GA paired with MILP [19], which combine evolutionary learning with CPSO [24,25], and SABAS [31] to improve swarm intelligence through adaptive control mechanisms. Furthermore, [33] offered a BA combined with an Asymmetrical Weighted Variational Method for exact path correction.

Other innovative contributions include GMOPSO-QL, which combines Gaussian Multi-Objective PSO and Q-Learning [34], and a dense neural network with TLBO [37], which utilizes deep learning for predictive planning. Classic graph-based search approaches have also been hybridized, as demonstrated in GWO with A* [44] and A* combined with DWA [47] for obstacle-aware trajectory planning.

Reinforcement learning has continued to improve through models like AD3QN [50], while evolutionary hybrids, such as Ant Colony Optimization combined with Particle Swarm Optimization (ACO-PSO) and Genetic Algorithm with Ant Colony Optimization (GA-ACO), have provided synergistic increases in path accuracy and search space coverage [51]. Other examples include HT-PSO [52], LODBO [53], and a genetic algorithm (GA) with Blockchain and Trust Models [54] for secure and efficient planning. Clustering-based techniques, such as Hybrid K-means with Local and Global TSP [55], perform well in multi-target routing.

These findings demonstrate that hybrid algorithms are not only durable and accurate but also adaptable in complex operational circumstances, making them an attractive avenue for future UAV path planning research.

## RQ-1

### Classification of UAV path planning

UAV path planning approaches can be generally grouped according to their strategic and operational elements. Methods are classified as global, local, or hybrid depending on the path-planning strategy. Global path planning calculates the entire route in advance, utilizing all available environmental information. In contrast, local path planning alters the course in real-time in response to dynamic impediments or environmental changes. Hybrid planning combines both methodologies to increase flexibility and efficiency.

The planning approaches differ depending on whether the UAV is operating in a static, dynamic, or known environment.

UAV path planning can be single-objective (optimizing one factor such as distance or energy) or multi-objective (balancing trade-offs between many criteria such as time, energy, safety, and risk).

Finally, depending on the number of UAVs, planning can involve a single UAV operating autonomously or a swarm of UAVs requiring coordination and communication to complete collaborative tasks.

**Fig. 1:**
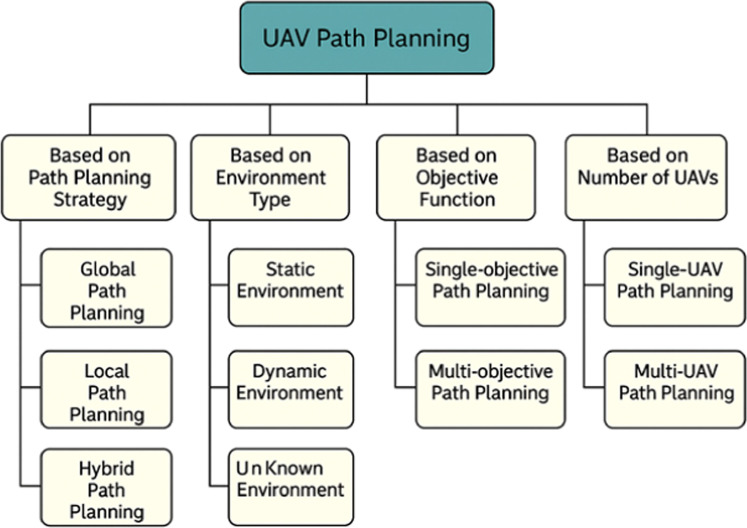
Classification of UAV path planning techniques.

Fig. 1 expressed that several of the categorization characteristics covered in this study may seem conceptually related, such as static vs known settings and global versus local planning. Instead of overlapping, these aspects are viewed as complimentary in this approach. While static and known environments refer to the temporal availability and completeness of environmental information, global and local planning are differentiated by the spatial extent of the decision-making process. Just as a local planner may rely on incomplete or changing knowledge, a global planner may work in a static or dynamic context. To guarantee conceptual coherence and clarity, this distinction is upheld throughout the categorization scheme.

### Global path planning

Different established methods have been developed for global path planning in static conditions where the topography and obstacles are known. These methods employ a range of algorithms specifically designed to enhance the path selection of Unmanned Aircraft Systems (UAS), ensuring both efficiency and safety.

Notable examples include the A* algorithm [62], a commonly used network search strategy that ensures the discovery of the shortest path; GAs [54], which use evolutionary principles to enhance path solutions gradually; PSO [63], which improves paths by simulating the collective movement of a swarm, inspired by the social behavior of birds; DE [64], which emphasizes global optimization through mutation and recombination strategies; and ACO [65], which imitates the Each of these algorithms offers distinct advantages in terms of computational efficiency, adaptability, and usefulness in various situations, making them critical tools for UAV global path planning.

### Local path planning

Local route planning is designed primarily for dynamic situations in which the UAV must adapt its direction in real time to avoid unexpected obstacles or respond to changing conditions. This technique promotes quick decision-making and computational efficiency, often at the expense of discovering the exact best answer. Furthermore, multi-UAV route planning adds another layer of complexity by requiring the synchronization of several UAVs to achieve a similar goal while avoiding collisions and optimizing their combined performance. Effective planning is crucial for operations that require the coordinated activities of multiple UAVs, mainly when operating in swarms [47].

To address local optimization issues, APF is enhanced further by adding an obstacle-generated gravitational function into the repulsive function [42]. Although the method is successful in simulation, it may face difficulties in real-world applications due to its sensitivity to parameter changes. A further improvement was demonstrated in the article, which utilized optimal control theory to fine-tune the potential field, resulting in fewer irregular routes [66]. However, the approach's reliance on precise environmental data may limit its flexibility in unpredictable situations. As a final step toward improved route stability, they introduced a novel boundary function in their work to limit the flight region of UAVs [67].

### Hybrid path planning

Table 4 shows that hybrid path planning algorithms use the benefits of two or more strategies to improve the overall performance of UAV trajectory optimization. These combinations try to address the shortcomings of solo approaches, such as delayed convergence, premature convergence, inadequate local optima avoidance, and adaptability, by combining their complementing advantages.

**Table 4:** Summary of hybrid algorithms applied in UAV path planning.

References	Hybrid Path Planning Algorithm	Purpose
[2,55,58]	HABC (Hill Climbing + ABC)	Combines global search (ABC) with local refinement (Hill Climbing) for better convergence.
[13]	VAINDIWPSO / IC-VAINDIWPSO (Improved PSO variants)	Enhances swarm coordination and obstacle avoidance under changing environmental conditions.
[17]	DFSCSO (Dispersal Strategy + Cuckoo Search)	Incorporates biological dispersal with CS to balance exploration and exploitation in dynamic terrains.
[21]	CPSO (Chaotic PSO)	Improves diversity and avoids premature convergence using chaotic maps in PSO.
[24,25]	Improved SABAS (Self-Adaptive BAS)	Enhances search accuracy through self-adaptive sensitivity adjustment for local and global path optimization.
[31]	Bat Algorithm + Weighted Variational Method	Improves convergence speed and accuracy by integrating adaptive variation into bat search behavior.
[50]	ACO + PSO, GA + ACO	Leverages ACO’s path construction with PSO/GA’s global optimization for multi-modal terrain.
[51]	HT-PSO (Hyperbolic Tangent PSO)	Uses a nonlinear activation function to enhance search dynamics and convergence behaviour.
[52]	LODBO (Landmark-guided Dung Beetle Optimization)	Uses landmarks to guide search, improving directionality in complex environments.
[54]	Hybrid K-means + Local & Global TSP	Uses clustering to segment environment and TSP for efficient intra- and inter-cluster path planning.
[19]	GA-QL (Genetic Algorithm + Q-Learning)	Uses Q-learning to adapt to environmental changes, with GA optimizing action choices.
[33]	GMOPSO-QL (Gaussian MOPSO + Q-Learning)	Enables learning-based path adjustment with swarm-based multi-objective optimization.
[34]	NN + TLBO (Neural Network + TLBO)	Learns from historical data and adapts route optimization using teaching-learning mechanisms.
[47]	AD3QN (Advanced Deep Double Dueling Q-Network)	Uses deep RL to make decisions in real-time under unknown and dynamic scenarios.
[53]	GA + Blockchain + Trust Model	Integrates secure decision-making with adaptive routing in dynamic or untrusted environments.
[37]	GWO + A*	GWO explores globally while A* performs precise local path refinement.
[44]	A* + DWA (Dynamic Window Approach)	A* plans a global path; DWA ensures dynamic obstacle avoidance during navigation.

Hybrid path planning solutions are increasingly combining heuristic and metaheuristic algorithms to improve performance in complicated UAV navigation tasks. For example, Hill Climbing paired with Artificial Bee Colony (HABC) [2,55,58] utilizes ABC's global search power and Hill Climbing's local refinement strength to provide more precise and optimal pathways, particularly in challenging terrain. Similarly, combining A* with the Dynamic Window Approach (DWA) [44] enables consistent global path development and real-time local obstacle avoidance, making it particularly useful in dynamic and congested situations.

Another effective combination is GWO + A* [37], which combines the global exploration capabilities of the Grey Wolf Optimizer with the heuristic strengths of A* for efficient and precise route design.

Incorporating reinforcement learning into hybrid optimization models has enhanced UAVs' ability to adapt to changing environments. GA-QL [19] and GMOPSO-QL [33] combine genetic and multi-objective particle swarm optimization methods with Q-learning, enabling UAVs to make real-time adaptive decisions based on environmental feedback. Similarly, AD3QN [47] combines deep reinforcement learning with advanced Q-network architectures to make real-time decisions in unknown settings.

The Neural Network paired with TLBO [34] improves UAV pathways by learning from previous trajectories and adjusting to dynamic changes in the terrain. These models enhance UAVs' ability to handle uncertainties and improve mission success rates in dynamic situations.

Other hybrid solutions aim to improve search space diversity and decision-making mechanisms. DFSCSO [17] employs a dispersal foraging method combined with Cuckoo Search to cover huge regions while conserving energy efficiently.

VAINDIWPSO and IC-VAINDIWPSO [13] incorporate inertia weight dynamics and chaotic behavior into PSO to improve swarm coordination while avoiding local optima. Improved SABAS [24,25], HT-PSO [51], and LODBO [52] use self-adaptive parameters, non-linear transformations, and landmark-guided behaviors, respectively, to improve path optimization and obstacle negotiating. GA + Blockchain + Trust Model [53] improves multi-agent or cooperative decision-making and secure communication between UAVs. Finally, Hybrid K-means + Local & Global TSP [54] enhances waypoint planning by utilizing intelligent clustering and multi-level route optimization.

### UAV path planning in static environment

In a static environment, all obstacles and terrain characteristics are established and known prior to the operation beginning. The placements of buildings, trees, terrain heights, and prohibited zones remain constant throughout the UAV's flight. Since no unexpected changes are anticipated during the mission, this setting allows for the pre-computation of the optimal flight path using deterministic or heuristic methods [25]. When we analyzed the considered environment in prior studies by comparing those that considered dynamic environments to those that considered static environments, we discovered that the majority of studies used a static environment. Specifically, 77 % of the surroundings were static, with only 23 % being dynamic [46]. This finding is validated by the level of complexity in dynamic situations, especially when using multiple UAVs [68].

Path planning in such contexts is primarily concerned with finding the shortest or most energy-efficient route while avoiding collisions. Algorithms such as A*, Dijkstra's, ACO, and PSO have been successfully applied in these cases due to their ability to explore known search areas thoroughly. The unchanging nature of the surroundings significantly decreases processing complexity [35], allowing for offline planning prior to takeoff.

Although static environment planning is simpler than dynamic path planning, it is critical in applications that require stable and predictable terrain and barriers, such as aerial surveys, agricultural monitoring, and infrastructure inspection.

### UAV path planning in dynamic environment

Table 5 present the summary of algorithms applied in UAV path planning in dynamic environments is complex due to the presence of shifting obstacles, changing targets, and fluctuating environmental conditions. Unlike static circumstances, dynamic environments require UAVs to adjust their trajectories in real time to maintain mission safety and efficacy. This adaptability is critical in complex and uncertain operational environments, including urban air mobility, disaster response, and military surveillance, where the environment can change unexpectedly during flight.

**Table 5:** Summary of algorithms applied in UAV path planning in dynamic environment.

Reference	Algorithms	Purpose
[19,33]	GA-QL, GMOPSO-QL	Reinforcement learning adapts to changing environments.
[44]	A* + DWA	Avoids moving obstacles using dynamic window.
[34]	NN + TLBO	Neural network adapts to dynamic paths from historical data.
[13]	VAINDIWPSO / IC-VAINDIWPSO	Handles swarm behavior under changing parameters.

In dynamic situations, UAVs must continually adjust their trajectories in response to changing obstacles, mission parameters, and real-time environmental conditions. Several hybrid algorithms have been developed to handle these complications effectively. For example, GA-QL and GMOPSO-QL [19,33] use reinforcement learning to help UAVs make intelligent judgments in changing settings.

The integration of A* with the DWA [44] improves real-time obstacle avoidance by integrating global path planning with reactive local maneuvering. Similarly, the combination of NN with TLBO [34] enables UAVs to learn from historical trajectory data and adjust to environmental changes dynamically. Furthermore, VAINDIWPSO and its improved chaotic counterpart IC-VAINDIWPSO [13] improve swarm-based path planning by altering velocity and inertia weights in response to changing factors. These hybrid approaches demonstrate how sophisticated algorithm combinations can significantly enhance UAV navigation in challenging environments.

### UAV path planning for unknown environment

UAVs operate in unknown areas with no prior understanding of terrain structure, barrier placement, or environmental dynamics. This is a substantial problem, as real-time decision-making and adaptability are critical to mission success. To overcome this, clever hybrid algorithms are frequently used, which combine global and local methods with learning capabilities.

In unknown or partially visible surroundings, UAV path planning becomes more challenging due to a lack of prior environmental data and the need for on-the-fly decision-making. In such cases, hybrid algorithms that combine learning and secure communication mechanisms have proven beneficial. For example, the Advanced Deep Double Dueling Q-Network (AD3QN) [47] allows UAVs to learn optimal paths in real time through deep reinforcement learning without the need for a predetermined map.

This versatility enables UAVs to respond quickly to unexpected obstacles or changes in the environment. Similarly, combining Genetic Algorithms with Blockchain and Trust models [53] enables secure, decentralized, and adaptive decision-making in settings where trust, privacy, and situational awareness are crucial. These strategies, when combined, enhance UAV autonomy, making them suitable for high-risk missions such as exploration, surveillance, and disaster response in unknown or hostile environments.

### Single-Objective path planning

Single-objective path planning is a fundamental strategy in UAV navigation that optimizes for a single criterion, such as decreasing distance, energy consumption, or flying duration. This strategy streamlines the planning process by focusing on a single, well-defined goal, resulting in faster computation and a more straightforward implementation. It is instrumental in organized or predictable contexts when mission objectives are clear and do not necessitate balancing various competing considerations. Single-objective techniques are commonly used in applications such as surveillance, area coverage, and point-to-point delivery jobs.

Table 6 provide the summary of algorithms applied in single-objective path planning. In single-objective path planning, the goal is to minimize a specified criterion, such as distance, energy, or time. Several well-known algorithms have been employed in this arena. The Dijkstra algorithm [43] is a deterministic method for finding the shortest path in a graph by thoroughly evaluating nodes, making it excellent for static environments with complete map information. On the other hand, the A algorithm* [44] incorporates heuristic estimation, which dramatically reduces computing costs while preserving optimal performance. Hill Climbing [58] is a greedy strategy for finding a close optimum, but it risks becoming caught in local minima. RRT [42] is helpful in high-dimensional or complex environments due to its randomized and quick exploration; however, it does not guarantee optimality.

**Table 6:** Summary of algorithms applied in single-objective path planning.

Algorithm	References	Purpose
Dijkstra Algorithm	[1,4,42,43,61]	Finds the shortest path in a graph deterministically.
A* Algorithm	[4,16,40,[42], [43], [44]]	Heuristic-based shortest path with lower computational cost.
Hill Climbing	[2,58]	Greedy method for local optimum path search.
RRT	[8,42]	Randomized approach for fast exploration in high-dimensional spaces.
Simulated Annealing (SA)	[45,46,49]	Probabilistic technique for finding a near-optimal solution.
Firefly Algorithm (FA)	[3,30,45,46]	Light intensity metaphor for finding optimal solutions locally.
Cuckoo Search (CS)	[17,30,45,49]	Inspired by brood parasitism for solution optimization.
Sine Cosine Algorithm (SCA)	[30,49]	Uses sine and cosine functions to explore solution space.
Whale Optimization Algorithm	[12,49]	Mimics bubble-net hunting strategy for global search.

Metaheuristic algorithms have also shown promising results in single-objective UAV path planning. SA [46] utilizes probabilistic jumps to circumvent local optima and seek near-optimal solutions. Nature-inspired techniques, such as the FA [30], utilize attraction based on light intensity to explore the solution space. In contrast, the CS algorithm [45] simulates brood parasitism to find better solutions, employing Lévy flights for enhanced global search efficiency. The SCA [49] employs trigonometric patterns for exploration and convergence, striking a balance between local and global search. Finally, the WOA [12], inspired by humpback whales' bubble-net hunting method, effectively simulates the encircling and attacking of prey to discover global optima. These algorithms provide powerful and adaptable tools for optimizing single-objective UAV path planning problems in both basic and complex settings.

### Multi-objective path planning

Multi-objective path planning is a crucial aspect of UAV navigation, as it necessitates the simultaneous consideration of multiple conflicting criteria. Unlike single-objective techniques that maximize only one parameter, such as distance or time, multi-objective planning considers multiple objectives, including energy efficiency, safety, flight time, terrain complexity, obstacle avoidance, and mission-specific limitations. The primary purpose is to identify a collection of optimal trade-offs from which the best path can be chosen depending on current mission priorities.

This method is particularly beneficial in real-world scenarios where UAVs must make informed decisions in the face of changing conditions and competing objectives. Multi-objective path planning enhances UAV mission adaptability, resilience, and overall efficiency by simultaneously addressing multiple objectives.

Multi-objective path planning aims to optimize two or more conflicting objectives simultaneously, such as minimizing path length, energy consumption, risk exposure, and flight duration while enhancing safety and data-gathering efficiency. Unlike single-objective techniques, multi-objective algorithms aim to generate a set of Pareto-optimal solutions, enabling decision-makers to make informed trade-offs based on mission-specific priorities. GA [21] is one of the most commonly utilized methods, as it employs evolutionary concepts to explore different paths while balancing objectives using fitness functions. ACO [26] is designed to simulate ant pheromone-guided search behavior and is ideal for dynamic and multi-objective optimization, particularly in routing issues. Similarly, the ABC algorithm [30] resembles bee foraging behavior and is ideally suited to balancing many criteria during search space exploration.

Other swarm intelligence systems, such as PSO [34], have demonstrated success in multi-objective path planning by simulating social and cognitive activities. BA [45], GWO [46], and DE [49] are also frequently used to investigate several solutions across objectives. These algorithms utilize adaptive parameters and co-evolution techniques to navigate complex landscapes efficiently. The strength of these techniques lies in their ability to maintain a diverse population of solutions while converging successfully toward trade-off fronts, making them ideal for UAV missions with varying operational limitations and objectives.

### Single UAV path planning

Single-UAV route planning involves calculating the optimal or most feasible trajectory for an individual unscrewed aerial vehicle to navigate from a starting point to a desired destination while adhering to various constraints. Obstacle avoidance, low energy usage, shorter travel times, and adherence to environmental or mission-specific standards are examples of such restrictions. The primary goal of single-UAV route planning is to create a safe, efficient, and executable path that allows the UAV to complete its task autonomously without relying on cooperation or communication with other drones.

In static situations, this planning can often be pre-computed using deterministic or heuristic techniques; however, in dynamic or unknown conditions, the UAV may use real-time or adaptive methods. Single-UAV planning is simpler than multi-UAV scenarios, but it still necessitates careful consideration of terrain characteristics, UAV kinematics, and onboard resource constraints. Efficient path planning enhances UAV autonomy and ensures mission success in various applications, including surveillance, delivery, inspection, and mapping.

The integration of machine learning and classical motion planning approaches has significantly enhanced single UAV path planning. Deep reinforcement learning techniques, such as Deep Q-Learning [1], Distributed DQN [38], DRL [41], and upgraded models like TD3 [23] and AD3QN [50], enable UAVs to learn successful navigation methods in complex and dynamic situations autonomously. Along with these, traditional path planning techniques such as Dubins paths [8], IPRM [10], and graph-based search algorithms, including D-Star and Voronoi algorithms [42], remain reliable frameworks for generating collision-free and feasible routes, particularly in structured or partially known areas.

Bio-inspired and swarm intelligence-based optimization methods have also significantly improved UAV path planning. Methods such as the PMMVO [11], RUPOA [12], and DTO [30] aim to optimize multiple-criteria objectives, including energy efficiency, obstacle avoidance, and mission restrictions. In addition, heuristic algorithms such as the BAS, Improved SABAS [31], and LODBO [53] have introduced novel approaches to enhance path planning speed and accuracy. Curve-based planning techniques, such as the Improved Bézier Curve with Receding Horizon Planning and SQP optimization [29], provide smooth trajectories that are compatible with UAV kinematic limitations.

Classical heuristic methods and metaheuristics continue to support these approaches by addressing issues such as real-time obstacle avoidance and processing efficiency. The Artificial Potential Field Algorithm [42] is commonly used for reactive collision avoidance; however, metaheuristics such as TLBO [37], GSA, and HS [49] provide strong frameworks for adaptive and efficient path planning in dynamic environments. This diverse set of algorithms exemplifies the evolving landscape of single UAV path planning, where adaptation, optimization, and real-time decision-making are crucial for effective navigation in complex and uncertain environments.

### Swarms of UAV path planning

Path planning for swarms of UAVs involves coordinating multiple autonomous aerial vehicles to achieve common goals, such as area coverage, target tracking, or search and rescue missions. In contrast to single UAV path planning, swarm-based planning requires addressing complex issues such as UAV collision avoidance, communication constraints, dynamic task allocation, and formation integrity. Effective swarm route planning must strike a balance between individual UAV autonomy and collective behavior to enable efficient coverage, resilience to failures, and flexibility in changing settings. This multi-agent coordination enhances the system's overall flexibility and scalability, enabling more sophisticated and resilient mission execution in complex and dynamic environments.

Path planning for swarms of UAVs employs a range of optimization and heuristic approaches to address the complexity of multi-agent coordination and dynamic environments. Traditional graph-based algorithms, such as Dijkstra [1] and A* [4], give fundamental shortest-path solutions but have scalability issues in large swarm activities. Nature-inspired algorithms, such as ACO [3] and ABC [2], are well-known for their decentralized decision-making and efficient exploration capabilities in swarm environments.

Evolutionary algorithms, such as the genetic algorithm (GA) [12], and swarm intelligence methods, including particle swarm optimization (PSO) [15], are effective in solving multi-objective optimization problems, including those related to collision avoidance and energy efficiency. More modern metaheuristics, such as the BA [30] and CS [17], enhance convergence and solution quality under challenging situations. Other intriguing algorithms include GWO [44], SA [45], FA [46], and RRT [8], which offer alternative approaches to navigating obstacle-rich and dynamic environments. Simple heuristics such as Hill Climbing [58] are occasionally used due to their computational simplicity. This heterogeneous algorithmic landscape highlights the importance of adaptive and scalable solutions in swarm UAV path planning, enabling coordinated, efficient, and resilient mission execution.

## RQ-2

### UAV path planning algorithms

**Fig. 2:**
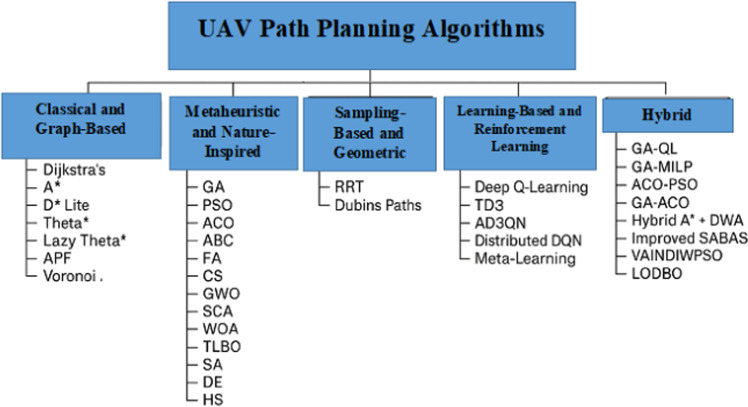
Summary of UAV Path Planning Algorithms.

The path planning problem is crucial to UAV navigation, with the goal of determining an optimal or near-optimal route from a starting point to a destination while avoiding obstacles, conserving energy, and adjusting to changing conditions. Fig. 2 shows that over the last few years, numerous algorithms for UAV path planning have been developed and modified, ranging from traditional deterministic methods to advanced metaheuristic, learning-based, and hybrid approaches. This section analyzes the main types of UAV path planning algorithms, focusing on their trends, changes, and applications as seen in current research from 2021 to 2025.

### Classical and graph-based algorithms

Classical algorithms, such as A* and Dijkstra's algorithm, remain important in UAV path planning, particularly in structured or known settings. These algorithms provide optimal solutions by methodically searching the state space using heuristics or edge weights. The A\* method was widely employed in studies [59] due to its balance of optimality and computing economy. Dijkstra's method and its modifications were also mentioned extensively [43] due to their usefulness in grid-based navigation systems.

Advanced variants, such as D*-Lite [14], Hybrid A* + DWA, and Lazy Theta* [47], provided flexibility for dynamic re-planning and smoother trajectories. These methods are well suited to indoor and GPS-denied situations, but they may struggle in high-dimensional or unstructured terrains without augmentation.

When the environment is completely understood and unchanging, classical and graph-based path planning algorithms like Dijkstra and A* produce optimum or nearly optimal solutions. For mission-critical applications, its deterministic nature guarantees consistent performance and repeatability. However, as the size and complexity of the environment grow, these approaches suffer from poor scalability and excessive processing costs. Furthermore, they are less appropriate for dynamic or unpredictable contexts due to their reliance on whole previous information. Therefore, in structured and static contexts, classical techniques are best suited for offline planning.

### Metaheuristic and nature-inspired algorithms

Metaheuristic algorithms have gained popularity due to their adaptability and ability to solve complex, non-linear, and multi-objective optimization problems in UAV routing. GA evolved as one of the most often used methodologies [21], frequently combined with Q-learning, MILP, or hybrid structures such as GA-ACO and GA-QL. PSO and its variations, such as Chaos PSO and HT-PSO, were also widely used [24] due to their swarm intelligence and global convergence capabilities.

Similarly, ACO has received considerable attention in studies [26], where it has been praised for its effectiveness in distributed search and suitability for terrain-based navigation. Other biologically inspired approaches, including ABC, BA, FA, and GWO [12], have provided lightweight yet effective alternatives, particularly in real-time UAV deployment scenarios. Emerging approaches, such as SCA and WOA, represent a growing trend in hybrid and adaptive swarm models.

Strong global search and exploration skills are demonstrated by metaheuristic and swarm intelligence-based algorithms, which allow them to successfully navigate complicated, non-linear, and obstacle-rich environments. These techniques are adaptable and may be used to solve multi-objective optimization problems with constraints related to safety, energy, and distance. However, they frequently need careful parameter adjustment, which may add computing cost, and they do not guarantee global optimality. Because of this, metaheuristic techniques are especially well suited for large-scale and partially understood situations where flexibility and variety of solutions are more crucial than rigorous optimality.

### Sampling-Based and geometric planning methods

In cases when deterministic approaches fail due to map size or unpredictability, sampling-based algorithms such as Rapidly Exploring Random Trees (RRT) have proven effective [8,42]. These algorithms rapidly produce feasible paths by randomly sampling the configuration space, making them excellent for large-scale and partially known settings. Enhanced approaches, such as Improved PRM (IPRM) [10] and Dubins' pathways [8], handle motion restrictions by incorporating curvature-aware planning, which is necessary for fixed-wing UAVs.

Furthermore, recent methods, such as Receding Horizon Planning with Improved Bézier Curves [29], offer smoother and dynamically adjustable trajectories that are suitable for real-time navigation.

### Learning-Based and reinforcement learning algorithms

Recent advances in machine learning have had a substantial impact on UAV path planning. Deep reinforcement learning techniques, such as Deep Q-Learning [1], Distributed DQN [38], and Advanced D3QN [50], offer robust frameworks for adaptive and real-time decision-making in uncertain situations. When sufficient training data is available, these models can outperform traditional methods.

Hybrid techniques, such as GA-QL [21], GMOPSO-QL [34], and TD3 [23], combine learning and optimization to improve flexibility and intelligence in UAV navigation. Deep neural networks [37], meta-learning [38], and teaching-learning-based optimization [37] all point to a trend toward more cognitive and data-efficient models that can be generalized across diverse missions and contexts.

In extremely dynamic and unpredictable contexts, learning-based path planning techniques especially those based on deep learning and reinforcement learning offer better flexibility. These methods are able to continually learn from feedback from the environment and make real-time policy adjustments. Despite these benefits, their real-time deployment on UAV platforms with limited resources may be limited since they usually require large training datasets and significant processing resources. As a result, learning-based approaches work best in dynamic settings when long-term performance gains and flexibility exceed training and computational expenses.

### Hybrid models

In addition to traditional techniques, researchers have developed specialized algorithms tailored to specific operational requirements. Custom approaches, such as VAINDIWPSO and IC-VAINDIWPSO [13], modify PSO to enhance convergence. Landmark-based methods, such as LODBO [53], and innovative optimization strategies, including DFSCSO [17], Improved SABAS [31], and DPREA [60], demonstrate a trend toward developing customized algorithms for challenging terrains and target tracking.

Region-based planners, such as those that employ NN and DENN [56], improve local route selection, particularly in clustered waypoint missions. Clustering techniques, such as K-means [26] and region-of-attraction estimates [57], enhance efficiency in multi-target or cooperative UAV scenarios.

The benefits of several algorithms are combined in hybrid path planning techniques to lessen the drawbacks of separate approaches. Hybrid approaches enable better convergence behavior, lower danger of local optima, and increased solution resilience by combining classical planners with metaheuristic or optimization-based methodologies. However, more processing demands and implementation difficulties might result from the increased algorithmic complexity. These methods work best in situations that require both flexibility and optimum solutions, especially in semi-dynamic settings.

## RQ-3

### Problem models for UAV path planning

Path planning for Unmanned Aerial Vehicles (UAVs) is a key challenge that involves determining optimal trajectories under various environmental, operational, and mission constraints. Recent research has presented a range of problem models to address this dilemma. These models vary in complexity, purpose, and assumptions and are frequently classified according to optimization approach, dimensionality, environmental awareness, and mission context.

### Single-Objective and multi-objective optimization models

Many researchers have initially concentrated on single-objective optimization models, which aim to maximize a specific performance indicator, such as path length, flight duration, or energy consumption. While these models are helpful for simple missions, they often fail to account for real-world complexity. As UAV missions become more demanding and complex, there is an increasing preference for multi-objective optimization models. These complex models balance various competing metrics, including energy consumption, collision avoidance, altitude stability, communication quality, and task completion success.

In practice, such models employ weighted or Pareto-based algorithms to balance conflicting goals within a set of practical restrictions that include battery life, range, payload limits, and agility. Multi-objective formulations often incorporate algorithmic methods, such as NSGA-II, MOPSO, or hybrid fuzzy-logic decision systems, to achieve optimal compromise solutions. These models are beneficial for surveillance, delivery, or multi-task missions when many performance objectives must be met [1,] [34,54].

### Real-Time and dynamic environment models

UAVs often operate in unpredictable, rapidly changing environments where real-time decision-making is essential. These dynamic environments may include varying landscape layouts, moving impediments (such as automobiles, birds, and other UAVs), and changing weather conditions. As a result, several models have transitioned from static path planning to dynamic and real-time models, allowing for adaptive path modifications during flight. These vehicles utilize onboard sensors, environmental monitoring, and high-speed processing units to scan their surroundings and recalculate safe and optimal routes continuously. Real-time models are critical in low-altitude flights, emergency response operations, and complex urban or rural situations where GPS data may be limited or unreliable.

These frameworks often employ techniques such as Model Predictive Control (MPC), online reinforcement learning, and reactive AI methods to achieve real-time responsiveness and environmental adaptability [14,17,31]..

### 3D Path planning and kinematic models

Traditional 2D models are insufficient for aerial robotics since UAVs are designed to travel in three dimensions. Thus, 3D path planning models are necessary to utilize UAV mobility effectively. These models consider vertical motion, height gradients, and spatial obstructions, necessitating precise altitude planning and energy-efficient vertical transitions. To mimic realistic UAV motion, many studies employ kinematic and dynamic constraints derived from UAV flight physics, including Newton-Euler equations, jerk minimization, yaw corrections, and pitch-roll limitations. Some sophisticated models employ energy functions based on aerodynamic drag, vertical climb rates, or thrust estimates. Topography data from digital elevation maps and LiDAR devices frequently aid real-time 3D modeling. This extensive modeling enables safe and efficient navigation in urban canyons, mountain passes, and obstacle-rich environments [10,11], and [27].

### Coverage path planning (CPP) and routing models

Aerial inspection, agricultural monitoring, and surveillance missions all require entire area coverage to ensure success. Coverage Path Planning models aim to cover a whole region with low redundancy and energy use. These models are frequently constructed using TSP, Vehicle Routing Problem, or grid-based decomposition methods. To improve performance, many studies propose hybrid frameworks that divide CPP into global and local planning components. TSP, for example, manages global routing across area patches, whereas local coverage inside each patch is achieved using systematic sweeps such as zigzag, spiral, or boustrophedon patterns. Some models use clustering methods to divide target areas into smaller subzones, which improves scalability and reduces computing complexity. These routing-based CPP models are crucial in applications requiring high-resolution data and consistent spatial sampling [16,26,56].

### Decision-Theoretic and learning-based models

Modern UAVs are increasingly utilizing sophisticated decision-making models based on decision theory and learning algorithms. These include Markov Decision Processes (MDPs), Partially Observable MDPs, and Reinforcement Learning (RL) methods, which enable UAVs to learn optimal behaviors through trial-and-error interactions in dynamic environments. Such learning-based frameworks enable UAVs to navigate in unknown conditions, adapt to changing objectives, and continually improve their performance over time. Furthermore, models for managing UAV trajectories in networked and resource-constrained environments have been suggested, which use mobility prediction techniques such as Gauss-Markov processes and energy-efficient control logic based on time-slotted decisions. These techniques are instrumental in applications that require long-term autonomy, such as disaster assessment, military reconnaissance, and adaptive monitoring of environmental variables [38,41].

By simplifying flexible decision-making, learning-based path planning techniques further alleviate the rigidity of traditional methods. For instance, planners based on reinforcement learning can improve robustness and flexibility by continually updating navigation strategies in response to dynamic impediments and unpredictable situations. Furthermore, by moving complexity to offline training, deep learning-assisted planners have been shown to lessen computing strain during online execution, making them appropriate for real-time UAV operations in dynamic circumstances.

### Hybrid models

To enhance flexibility and accuracy in UAV path planning, researchers have developed hybrid models that integrate multiple modeling techniques and algorithms. These models aim to overcome the limitations of individual procedures by combining the strengths of multiple methodologies. For example, combining MPC with metaheuristic algorithms, such as ACO or PSO, can improve convergence speed, path quality, and energy efficiency. Some hybrid models combine physics-based flight dynamics with environmental modeling, which employs Lagrangian mechanics to represent damping, turbulence, and energy dissipation. More recent advancements include the use of blockchain technology for secure data exchange and trust-based systems to prevent data manipulation or signal spoofing during multi-UAV missions. Such cross-domain hybrid models are vital for solving complicated tasks that require coordination, adaptability, and data integrity [54,57].

Numerous research show that hybrid path planning techniques successfully get beyond the drawbacks of traditional methods. For example, it has been demonstrated that combining Ant Colony Optimization with Model Predictive Control reduces local-optima entrapment while preserving trajectory feasibility and smoothness. In a similar vein, hybrid frameworks that combine metaheuristic optimization with graph-based planners improve global path optimality while maintaining environmental flexibility. These examples show how hybrid approaches surpass the capabilities of solo classical planners in balancing solution quality and adaptability.

### Environment-Specific and constraint-based models

Many path-planning models are designed for specific operating contexts, resulting in unique restrictions and mission needs. These habitats include hilly terrains, offshore platforms, urban canyons, deserts, and forested areas. Each environment presents unique challenges, including line-of-sight obstructions, GPS signal loss, wind disturbances, and restricted flight paths. Constraint-based models explicitly incorporate these limitations into the optimization framework by establishing limits on altitude, battery life, communication quality, and obstacle density. Some models employ environmental maps to represent threat zones or no-fly zones. Recent research has proposed cooperative multi-objective formulations that balance mobility, terrain avoidance, and communication dependability. Such models are critical for deployment in areas such as border patrol, sea rescue, and infrastructure inspection [10,17,31,60].

### Emerging trends and high-dimensional models

As UAV systems transition toward swarm-based operations and mission-scale expansion, the demand for high-dimensional path planning models increases. These models handle a greater number of variables and agents, which increases computational demand and coordination complexity. New developments emphasize scalable optimization approaches, including hierarchical planning, swarm intelligence, and parallel processing. Graph-based abstraction, distance-aware dynamic coefficients, and spatial decomposition are among the techniques employed to minimize computational time and enhance solution quality. In high-dimensional settings, robust communication protocols and decentralized coordination frameworks are required to avoid collisions and maintain task efficiency. These models serve as the foundation for the next generation of autonomous UAV networks, which can coordinate in real-time across broad and diverse mission environments [28,52], and [61].

## RQ-4

### Emerging challenges

Recent research has identified various growing obstacles that impede the development and deployment of effective UAV path planning systems, particularly in dynamic, real-time, and resource-constrained contexts. These challenges are broadly classified below, with supporting credentials for each.

### Computational and real-time constraints

Modern UAV path planning algorithms must operate under strict real-time constraints while handling increasing computational complexity. Advanced optimization, hybrid, and learning-based methods often require significant processing power, which may limit their deployment on resource-constrained UAV platforms. Achieving a balance between solution quality and real-time feasibility therefore remains a critical challenge, particularly in dynamic and mission-critical applications.

### Environmental uncertainty and sensing limitations

Uncertainty in environmental modeling, dynamic obstacles, and imperfect sensor data poses significant challenges for reliable UAV path planning. Limitations in sensing accuracy, communication delays, and incomplete environmental knowledge can degrade planning performance and lead to suboptimal or unsafe trajectories. Developing robust algorithms capable of handling uncertainty and sensor imperfections remains an open research problem.

### Energy, and system integration

As UAV missions become more complex, scalability to large environments and multi-UAV systems introduces additional challenges. Energy constraints, communication reliability, and integration with onboard control and perception systems must be jointly considered to ensure practical deployability. Addressing these interdependent factors is essential for real-world UAV path planning applications.

## RQ-5

### Discussion

The landscape of UAV route planning has undergone significant changes, but some persistent gaps and research opportunities remain. In Table 7 our survey reveals that while traditional approaches, such as PSO, ACO, and GA, perform well in idealized circumstances, they struggle in real time, particularly in dynamic and uncertain environments. Hybrid models that combine Model Predictive Control (MPC), fuzzy logic, and reinforcement learning offer potential adaptability; however, their complexity and tuning issues hinder scalability in large-scale or multi-UAV operations. Problem modeling is also important many contemporary frameworks have moved away from deterministic techniques and toward probabilistic, fuzzy, or hybrid models, which better accommodate real-world uncertainties such as sensor noise, wind variability, and missing terrain data.

**Table 7:** Comparative summary of UAV path planning dimension.

Feature	Key Approaches / Findings	Strengths	Limitations	Suitable Scenarios	References
**Techniques**	Offline, Online, Hybrid	Predictability, Adaptability, Hybrid	Offline: not adaptive; Online: high computation; Hybrid: complex design	Static, Dynamic, and Hybrid	[6,9,14,18,20,23,28,31,36,39,44]
**Algorithms**	PSO, ACO, GA, MPC, RL, Fuzzy, ABC, Cuckoo Search, Hybrid Models	Metaheuristics: global exploration; MPC: real-time control; RL: learning; Fuzzy: interpretability	Local optima, convergence instability, resource-intensive	Dynamic terrain, multi-objective, real-time missions	[7,13,16,21,22,25,30,36,41,45,48,60,63,64]
**Problem Models**	Deterministic, Probabilistic, Fuzzy, Hybrid	Deterministic: simple; Probabilistic: uncertainty handling; Fuzzy: flexible decision logic	Unreliable under sensor error or data sparsity	GPS-denied, partially known, urban, cluttered	[5,10,11,15,27,29,33,42,53,57,59]
**Optimization Goals**	Time, Distance, Energy, Safety, Coverage, Connectivity	Customizable by mission; scalable in design	Multi-objective conflict, needs trade-off resolution	Surveillance, tracking, search-and-rescue, delivery	[4,8,12,17,24,26,34,38,41,47,49,51,55,61,65]
**Challenges**	Real-time adaptation, energy limits, dynamic obstacles, multi-UAV coordination, security, uncertainty, sensor faults	Identify research gaps; drive robust, adaptive design	Computation, bandwidth, synchronization, hardware limits	Swarms, real-world deployments, long endurance tasks	[3,19,31,35,37,40,41,43,46,50,52,54,56,58,60,62]

Despite these advancements, current algorithms sometimes fall short of balancing energy economy, resilience, and computational cost, particularly in GPS-denied or communication-constrained settings. Multi-objective optimization, although more popular, lacks standardized trade-off frameworks, which limits its practical utility. Domain-specific customization (e.g., for surveillance, mapping, or delivery) often requires specialized models and algorithmic tuning, thereby complicating generality. Finally, difficulties such as swarm coordination, cybersecurity, real-time responsiveness, and terrain-aware 3D planning continue to challenge the effectiveness of current methodologies. The field is thus at a crossroads where cognitive hybridization, contextual adaptability, and mission-specific design must all come together to power the next generation of UAV autonomy.

The comparative study in Table 1 demonstrates the complex nature of UAV path planning, as well as the interaction of algorithmic methodologies, environmental parameters, and mission-specific objectives.

Starting with path planning techniques, offline solutions are effective in predefined static contexts but ineffective in dynamic settings. Online approaches enable UAVs to adapt to real-time data and environmental changes, but they require large processing resources, as detailed in [31] and [36]. Hybrid approaches try to incorporate the best of both, but they sometimes require complicated architecture and synchronization, making them unsuitable for restricted onboard systems.

In terms of algorithms, metaheuristic techniques such as PSO, ACO, and GA are popular due to their strong global search capabilities. However, they typically experience convergence challenges and can become locked in local optima if not appropriately hybridized or tuned [21,36]. RL allows for adaptation through trial-and-error learning, making it suitable for dynamic and unpredictable contexts, but its high training cost limits its use in real time [41]. MPC ensures adherence to limitations and dynamic updates, although it is computationally demanding. Hybrid techniques are gaining popularity for addressing these trade-offs, however generalization remains difficult [60].

Another important factor to consider is problem modeling. Deterministic models are best suited for structured, predictable contexts, but probabilistic and fuzzy logic-based models are more resilient to uncertainty [29,53]. However, they are strongly reliant on dependable sensor data and robust estimation procedures, which may be unavailable in GPS-denied or signal-degraded contexts [57].

The optimization objectives differ greatly by application. Many studies attempt to reduce energy consumption or mission duration while ensuring safety and connection [41]. The issue arises when multiple goals clash, such as shortest path vs. energy-efficient path, resulting in optimization trade-offs that are difficult to resolve without multi-objective algorithms or hybrid models [51,55].

Finally, the rising issues highlight the gaps between theoretical models and real-world applications. Real-time decision-making, dynamic obstacle avoidance, and communication dependability are significant challenges [31,41]. Problems with coordination and bandwidth are typical in multi-UAV systems, especially in remote or hostile areas [54]. Spoofing and hijacking are growing security vulnerabilities as systems become more complex and autonomous. Furthermore, integrating several algorithms and customizing them for certain domains adds a further layer of difficulty [60].

## Future directions

Future UAV path planning research should focus on models that integrate real-time flexibility and multi-objective optimization to address more complicated mission conditions. With the rise of smart cities, disaster zones, and autonomous logistics, UAVs must function in a variety of dynamic and limited contexts. Thus, implementing adaptive control methods based on onboard sensors, edge computing, and lightweight AI will be critical.

Furthermore, hybrid models that combine machine learning, game theory, and control theory may be better suited to dealing with uncertainty in communication and environmental dynamics. Swarm-based path planning requires scalable, decentralized algorithms that can preserve network connectivity, minimize collisions, and achieve shared goals within resource constraints.

Blockchain-based trust frameworks, quantum-inspired optimization, and neuromorphic computing are emerging technologies that have the potential to enhance security, computational efficiency, and real-time learning capabilities. Furthermore, the integration of UAVs with 5 G and the Internet of Things (IoT) infrastructures enables cooperative multi-agent missions in smart environments. Integrating energy harvesting strategies, dynamic airspace policies, and ethical frameworks will all influence the future evolution of autonomous UAV path planning systems.

## Conclusion

This survey thoroughly examined a wide range of UAV path planning models, categorizing them into eight major categories based on their optimization objectives: environmental adaptability, real-time responsiveness, kinematic complexity, coverage requirements, decision intelligence, hybridization, and environmental constraints. It was discovered that while single-objective models are computationally simple, they fall short in dynamic mission contexts. Multi-objective techniques enable a more balanced trade-off between competing objectives, such as energy efficiency, flight time, and obstacle avoidance. Furthermore, the increasing demand for real-time adaptation has led to a greater emphasis on dynamic replanning and sensor-integrated models that enable real-time decision-making.

Three-dimensional path planning and kinematic modelling have gained popularity due to their ability to reflect the realistic dynamics of UAVs accurately. In contrast, CPP and routing models are still evolving in large-scale applications, including agriculture, surveillance, and inspection. Learning-based and decision-theoretic models have helped UAVs become more autonomous and adaptable in uncertain contexts. Hybrid and environment-specific models enhance UAV resilience and security, enabling them to be tailored for specialized missions, including urban navigation, offshore surveillance, and collaborative operations.

Despite these developments, the literature indicates that most contemporary models suffer from scalability issues, computational burden, and difficulties in integrating diverse mission requirements. Furthermore, energy-aware planning, secure communication, and swarm coordination are only partially addressed concerns. The debate brings together insights from 68 sources, enabling scholars and practitioners better to appreciate the strengths and limitations of current methodologies and identify the most effective models for mission-specific needs. Overall, this survey provides a knowledge baseline that connects theoretical foundations to practical applications in the UAV path planning arena.

## Ethics statements

MethodsX has ethical guidelines that all authors must comply with.

## If your work involved data collected from social media platforms

Our work did not involve data collected from social media platforms.

## CRediT author statement

Muhammad Nafees: Conceptualization, Methodology, Software, Validity Tests, Supervision, Writing- Draft preparation, Reviewing, Editing, Validation and Data Curation. Tamkeen Syeda and Amjad Ali: Verifying writing- Original Draft preparation, Reviewing, Editing and Validation. Hira Farman and Muhammad Tayyab Visualization and Investigation.

## Supplementary material and/or additional information [OPTIONAL]

“None”.

## Declaration of competing interest

The authors declare that they have no known competing financial interests or personal relationships that could have appeared to influence the work reported in this paper.
